# A Review on the Role of Endogenous Neurotrophins and Schwann Cells in Axonal Regeneration

**DOI:** 10.1007/s11481-021-10034-3

**Published:** 2021-11-29

**Authors:** Samyak Pandey, Jayesh Mudgal

**Affiliations:** Department of Pharmacology, Manipal College of Pharmaceutical Sciences, Manipal Academy of Higher Education, Manipal, Karnataka India 576104

**Keywords:** Neurotrophic factors, Schwann cells, Regeneration, Brain-derived neurotrophic factor, Pituitary adenylyl cyclase-activating peptide, Plasticity

## Abstract

Injury to the peripheral nerve is traditionally referred to acquired nerve injury as they are the result of physical trauma due to laceration, stretch, crush and compression of nerves. However, peripheral nerve injury may not be completely limited to acquired physical trauma. Peripheral nerve injury equally implies clinical conditions like Guillain-Barré syndrome (GBS), Carpal tunnel syndrome, rheumatoid arthritis and diabetes. Physical trauma is commonly mono-neuropathic as it engages a single nerve and produces focal damage, while in the context of pathological conditions the damage is divergent involving a group of the nerve causing polyneuropathy. Damage to the peripheral nerve can cause a diverse range of manifestations from sensory impairment to loss of function with unpredictable recovery patterns. Presently no treatment option provides complete or functional recovery in nerve injury, as nerve cells are highly differentiated and inert to regeneration. However, the regenerative phenotypes in Schwann cells get expressed when a signalling cascade is triggered by neurotrophins. Neurotrophins are one of the promising biomolecules that are released naturally post-injury with the potential to exhibit better functional recovery. Pharmacological intervention modulating the expression of these neurotrophins such as brain-derived neurotrophic factor (BDNF) and pituitary adenylyl cyclase-activating peptide (PACAP) can prove to be a significant treatment option as endogenous compounds which may have remarkable innate advantage showing maximum ‘biological relevance’.

**Graphical abstract:**

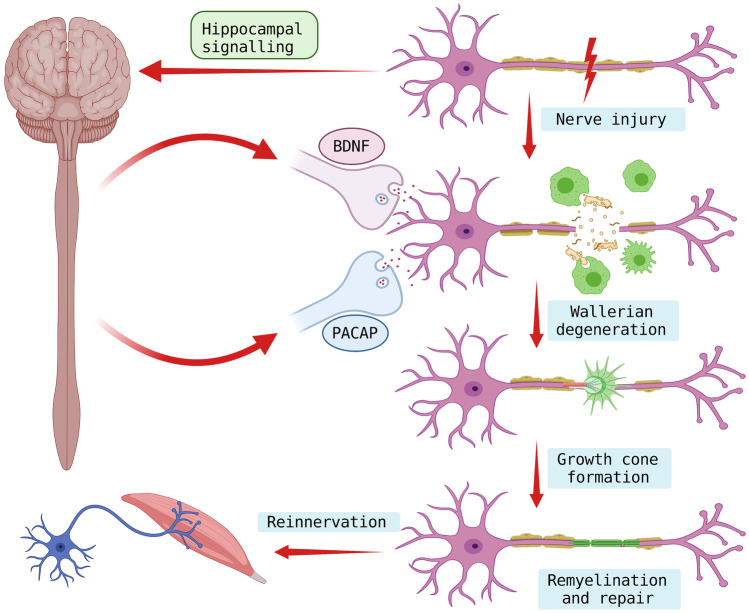

## Introduction

Neurons have the inherent ability to regenerate right from the primitive stage of neural development. As the neuronal cells mature, their ability to regenerate get highly suppressed. This suppression is noteworthy as they are highly specialized and differentiated cells. Mature nerve cell naturally lacks the microenvironment required for cell division or any type of growth other than structural maintenance. However, the dormancy for regeneration is terminated when a nerve cell is damaged and the regeneration phenotypes are expressed, this is made possible with the help of complex signalling within the cell. The regenerative phenotypes within the neurons are expressed or replenished with the help of endogenous neurotrophins. Nerve repair and regeneration post-injury have always been under trial in neuro and regenerative medicine. Although peripheral nerves are capable of regenerating to some extent, poor recovery is observed in patients. Nerve injury adds disability to an individual due to the indisposed motor and sensory functions. Being the most involuted and delicate system of the body, the nervous system is highly susceptible to various degrees of damage ranging from physical trauma to nerve degeneration. Unfortunately, due to the extreme complexity and dormancy of neurons, repair and regeneration to regain the functionality of neurons become a challenge. Since there is no ideal therapeutic strategy for nerve repair and regeneration, understanding the molecular mechanism for neuronal repair becomes a fundamental priority. Recent advances and pieces of evidence have made endogenous molecules like Brain-derived neurotrophic factor (BDNF) and pituitary adenylate cyclase-activating peptide (PACAP) the substance of choice for the therapeutic management of peripheral nerve injury (Pettersson et al. 2014). Neurotrophins have great potential to regulate disaster management to counter cellular insults, this ability of neurotrophins provides promising hints as to future therapeutic options for nerve injury. BDNF and PACAP are among the remarkable neurotrophins aiding cell survival and axonal recovery (Botia et al. 2011; McGregor and English 2019; Morio et al. 1996; Waschek et al. 2006; Yang et al. 2014). Synthesis of BDNF is known to be triggered in the hippocampus during physical exercise however, injury to nerve cells also acts as a trigger. BDNF activation promotes cell survival, differentiation and neurogenesis (De la Rosa et al. 2019; Liu and Nusslock 2018; Middlemas 2007; Ortiz-López et al. 2017; Seifert et al. 2010). PACAP is proven to act as neuroprotective in damaged neurons (Baxter et al. 2011; Kaneko et al. 2018; Morio et al. 1996; Tamas et al. 2012; Waschek et al. 2006). Along with the endogenous neurotrophins, the main execution role is performed by the Schwann cell (SC) which is an integral part of the peripheral nerve cell. Schwann cells are known to play a key role in myelination and providing trophic support to the neuron. Although basal functions of Schwann cells are more discussed, their ability to act during neuronal damage is neglected. Schwann cells are found to show a great amount of activity in an injured neuron, from clearing the cellular debris to adding new fibres to the damaged ends. They are assisted by the cells of innate immunity and fibroblast aiding the process of regeneration when triggered by specific signalling molecules. With the purpose to understand the science of axonal regeneration, it is imperative to understand the molecular mechanism of regeneration. This article presents the recent key updates in understanding the molecular mechanism and role of vital neurotrophins: BDNF and PACAP in Schwann cell-mediated axonal repair and regeneration (Gordon 2009; Maugeri et al. 2020; Yi et al. 2016).

## Nerve Injury, Classification and Regeneration

Peripheral nervous system (PNS) neurons are greatly inclined towards regeneration as compared to central nervous system (CNS) neurons. CNS neurons fail to regenerate as compared to PNS neuron (Huebner and Strittmatter 2009), since the cellular clean up in CNS is slow, and the absence of the Schwann cells make them an unfavourable candidate for the regeneration process. On the other hand, PNS neurons show extensive clearance with the help of macrophages and are added with Schwann cells (Kang and Lichtman 2013). Neurons can regenerate only if the cell body (soma) is intact, if the soma is damaged the neuron cannot regenerate naturally, however, the axon can regenerate in the case where damage to a segment of the neuron is away from the soma.

Regeneration of peripheral neurons relies on the type and extent of damage caused. Notably, two classifications of nerve injury exist as described by Seddon and Sunderland (Seddon 1943; Sunderland 1951). According to Seddon’s classification, there are 3 broad categories of nerve injury with increasing order of damage severity i.e. i) Neuropraxia, ii) Axonotmesis, and iii) Neurotmesis (Seddon 1943). On the other hand, Sunderland classifies nerve injury as five different grades based on the severity of the injury where Neuropraxia is designated as a grade-I injury while Axonotmesis is assigned grade-II to IV depending on the severity of the damage and grade-V denotes Neurotmesis (complete nerve transection) (Sunderland 1951).

Overall, *neuropraxia* or grade I injury is the mildest form of nerve damage with reversible conduction block due to mild axon or myelin compression without any anatomical damage to the protective layers of the neuron. *Axonotmesis* is demyelination and axonal disconnect but with intact endoneurium, however, the segment of the lost axon can regenerate gradually. The most severe form of nerve injury is *Neurotmesis* or grade-V injury. Demyelination, axon loss and protective layer disruption are the characteristic features of Neurotmesis. Grade-V injury indicates complete nerve transection and the injury is almost irreversible. The regrowth completely depends on the damage to the different protective layers, endoneurium damage shows fair growth; while perineurium damage shows poor growth. There is no growth observed if epineurium is damaged. For a neuron to regenerate without any pharmacological intervention the cut ends of the axon must be aligned and the distance between the two ends must be optimum for regeneration.

Regeneration of nerves is a critical and slow process with multiple stages of complex molecular arrangement. The process of regeneration begins from the proximal end of the neuron with the help of regenerative sprouts called fibrils/growth cones. These growth ends are aided with actin and myosin protein upregulation assisting the fibrils to the distal end of the neuron guided by Schwann cells. When Schwann cells align in the neurilemma tube they synthesize various nerve growth factors, which accelerates the process (Dent and Gertler 2003; Huebner and Strittmatter 2009; Kang and Lichtman 2013). The full cylinder axis is formed approximately in 3 months; however, 20% of the original diameter is lost in the process even after complete recovery. Remyelination takes about 12 months to regain complete integrity (Jessen and Mirsky 2019; Menorca et al. 2013; Sulaiman and Gordon 2013). To achieve a completely functional nerve, the neuron must undergo three key processes namely: Wallerian degeneration (WD), axonal regeneration and end-organ reinnervation.

## Post-injury Changes in Neuron

**Fig. 1:**
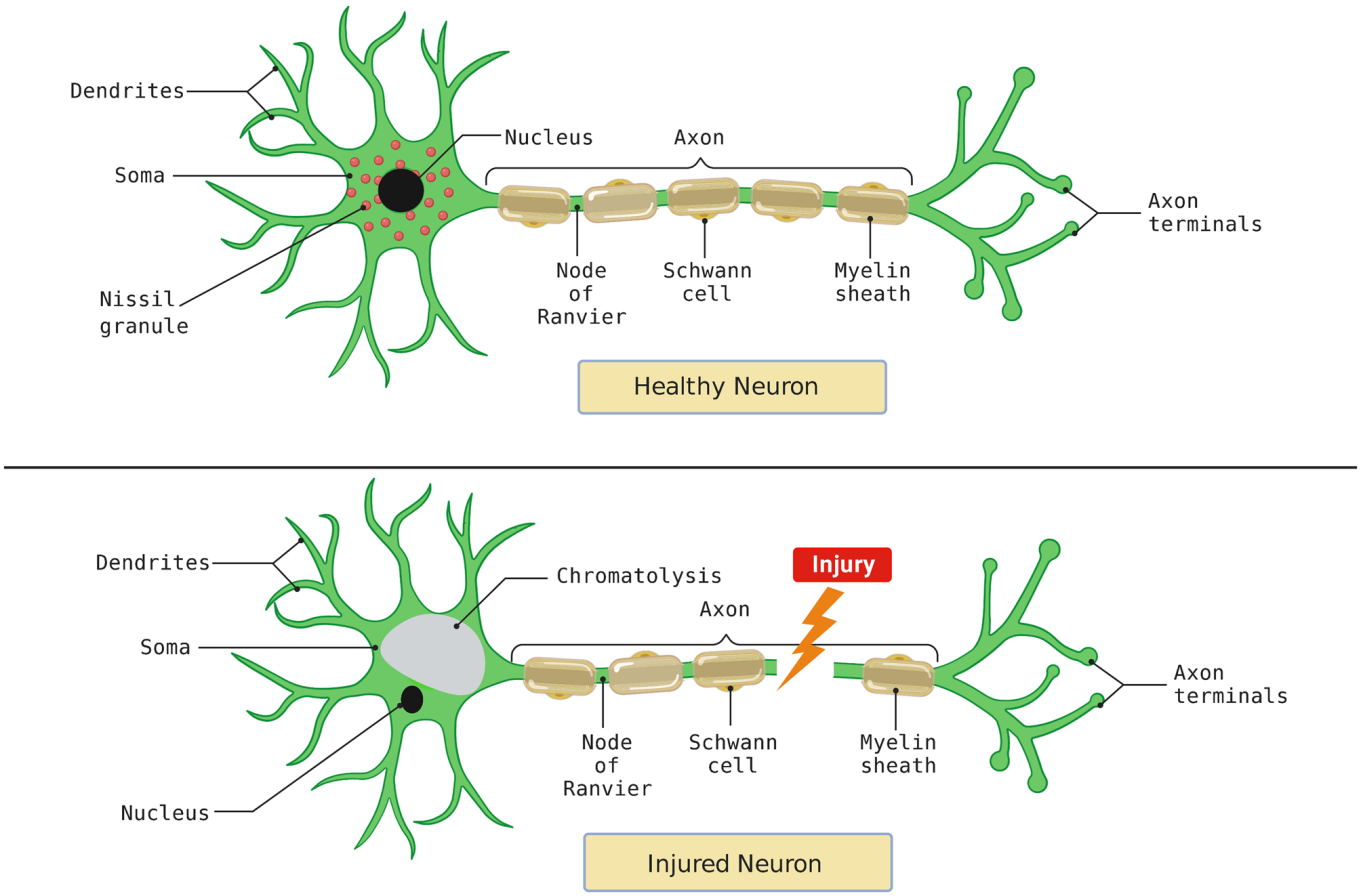
Summarised representation of post-injury events observed in neurons compared to a healthy neuron. A healthy neuron marks the presence of Nissl’s body, intact myelin and axon with nucleus occupying a central position in the cell body (created with BioRender.com)

Nerve cells respond uniquely to axonal injury following characteristic molecular and morphological changes which are together termed as retrograde neuron reaction or axon reaction (Ambron and Walters 1996). Major changes are expressed by the soma (cell body of neuron) exhibiting the characteristic chromatolysis following dispersion of Nissl bodies, increase in the size of cell body initially, followed by atrophy. The disappearance of major cell organelles is seen including Nissl’s granules or dark basophilic rough endoplasmic reticulum. The shifting of the nucleus from the central to the peripheral position is collectively referred to as central chromatolysis. As a result of chromatolysis, the cell shows the absence of all vital organelles dissolved in the matrix in the cytoplasm as chromatolytic / fragmented mass (Fig. 1). These changes are observed mainly between 1–3 weeks of axonal damage (Ambron and Walters 1996; Jessen and Mirsky 2019; Zochodne 2012). Along with soma the axonal region experiences major changes including demyelination of the damaged stumps. Myelin which is closer to the cut end degenerates while on the distal end, the entire distal segment degenerates in the process of recovery. Degeneration of the distal end is a complex procedure involving several immune cells for cleaning up the debris. An injured neuron exhibits relocation of the nucleus to the periphery closer to the cell membrane, chromatolysis of Nissl’s body is seen with chromatolytic mass (shown in grey colour, Fig. 1). The post-injury changes observed in neurons are protective and constructive in nature following a step towards functional recovery.

## Survival and Functional Role of Schwann Cell Post-injury

Schwann cells (SC) synthesize myelin in a normal cell, however, in the event of neuronal damage new phenotype is acquired ceasing the myelination process. This leads to the formation of bands of bungers. SCs produce cytokines that will recruit macrophages promoting clearance of damaged axonal debris, and synthesis of neurotrophic factors (Dent and Gertler 2003; Jessen and Mirsky 2019; Qu et al. 2021). SCs are present as myelinating and non-myelinating cells (Remak cells) in peripheral nerve fibres. Myelinating SCs are found surrounding axons of greater diameter or larger axons conversely Remak cells are found surrounding axons of lesser diameter or smaller axons. In a matured myelinated SC, the nucleus is found in the periphery of the cell while in the case of remak cell, the nucleus occupies a central position. A single myelin bundle encloses only one axon, Remak bundle can enclose multiple axons, when c-fibre axons are enclosed together it is called a Remak bundle. The SCs have an unusual ability to transform themselves into immature cells by changing their phenotype in response to injury, this property of SCs is known as the phenomena of plasticity. Plasticity is practised when cells detect the absence of contact with the axon in the event of injury. Myelinated SCs play a crucial role in maintaining the normal function of neurons by providing them trophic support and factors responsible for their maintenance (Boerboom et al. 2017; Jessen et al. 2015; Zochodne 2012). However, in the event of injury, non-myelinating SCs (Remak cells) undergo de-differentiation resulting in the formation of myelinating Schwann cells. These non-myelinating SCs function to achieve plasticity and support the process of nerve regeneration, like myelinating SCs (Jessen and Mirsky 2019; Monje 2020). The process of axonal regrowth is marked by elevated expression levels of BDNF like neurotrophins, trophic support and signalling triggers which are provided by Remak cells after dedifferentiation of SCs (Jessen and Mirsky 2016; Jessen et al. 2015; Jessen and Mirsky 2019). SCs helps in neuronal cell survival and elongation of axon in case of injury by upregulating GDNF, BDNF, NT-3, NGF and VEGF. SCs show a cascade of effects in response to injury by releasing tumour necrosis factor-α (TNF-α), interleukins like IL-1α, IL-1β and monocyte chemotactic protein-1 (MCP-1). The release of MCP-1 attracts macrophages to the site of injury; these macrophages play a key role in WD. After WD of the damaged axon, macrophage helps in downregulating the pro-inflammatory cytokines and upregulating the anti-inflammatory cytokines Jessen and Mirsky 2016; Rawji et al. 2016; Rotshenker 2011). Once WD is achieved, it is important to bring down the levels of pro-inflammatory cytokines, failure to stop inflammation results in non-functional or no recovery (David et al. 2015; Dubový et al. 2013; Siqueira Mietto et al. 2015). In a study (Siqueira Mietto et al. 2015), the anti-inflammatory role of IL-10 was demonstrated with the help of IL-10 null mice with crush nerve injury. Following an absence of IL-10, there was no stable regeneration and functional recovery observed. Therefore, to counter the inflammatory environment created pre-WD, IL-10 expression proves vital as an anti-inflammatory cytokine.

## Myelin: from Conduction to Growth Inhibition

Myelin is predominantly found in PNS which facilitates saltatory conduction in large axons increasing the velocity of conduction of nerve impulse down the axon terminal. Myelin is composed of 70% lipid; sphingolipids, cholesterol, saturated long-chain fatty acids, glycolipids are other components of myelin (Siegel and Agranoff 1999). Myelination is controlled by several cellular factors, peptides and proteins, out of them there are 4 important known factors responsible for synthesis, maintenance and regulation of myelination, namely myelin protein zero (MPZ), Krox-20, maltose-binding protein (MBP) and myelin-associated glycoprotein (MAG). MPZ belongs to the immunoglobin superfamily and contributes up to 60% of total myelin protein (Suter and Martini 2005). It is a transmembrane supermolecule providing compactness to axons using cholesterol post myelination. Krox-20 (also known as Egr2) is a pro-myelin transcription factor, controlling genes crucial for late-stage axonal myelination (Ghislain and Charnay 2006). MBP is found in the peripheral region of the cell membrane and assist myelin arrangement. Injury to axon results in reversal of all somatic processes within the neuron impairing synthetic functions. This is marked by the downregulation of pro-synthetic factors: MPZ, MAG, Krox-20 ceasing the process of myelination. Expression levels of these pro-myelin factors are seen elevated again when the axon is successfully regenerated triggering remyelination across the nerve tract (Jessen et al. 2015; Suter and Martini 2005; Svennigsen and Dahlin 2013; Zochodne 2012). Expression levels of glial fibrillary acidic protein (GFAP), neural cell adhesion molecule (NCAM) and p75 neurotrophin receptor (p75^NTR^) are found characteristically elevated during axonal injuries, however, GFAP levels are found to be more promising and accurate in nerve injuries (Gonçalves et al. 2019; Notturno et al. 2009; Thornton et al. 2005). As myelin forms part of a matured and intact neuron when damaged, it acts as a hindrance to the regeneration process by accumulating debris and expressing certain growth inhibitory factors within myelin. Inhibitors like Nogo, MAG, oligodendrocyte myelin glycoprotein (Omgp) break down the newly formed growth cone terminating axonal growth (Filbin 2003; Hannila and Mellado 2017; Maugeri et al. 2020).

## Demyelination, Axon Bridging, Myelination: a Functional Neuron

**Fig. 2:**
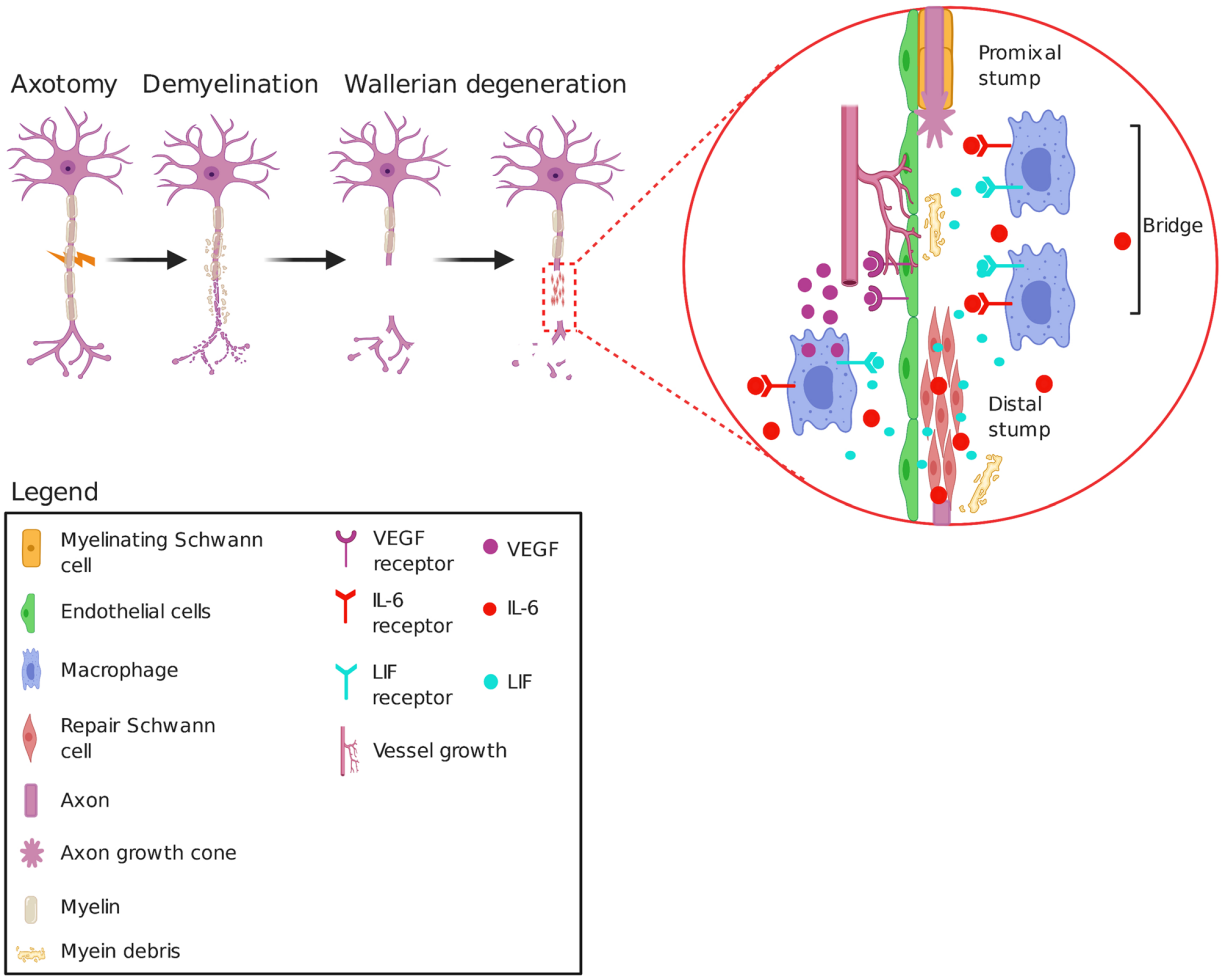
Diagram depicting the mechanism of post-injury repair and regeneration process guiding the assembly of the axonal bridge. The figure illustrates the triple response (demyelination, WD, axonal bridge assembly) to transactional injury (created with BioRender.com)

Other than the myelinating and non-myelinating SCs, repair SCs play a vital role in the regeneration of the axon. Repair cells remain inert and are active in basal conditions however, they are expressed only in damaged cells (Jessen and Mirsky 2016). These repair cells show characteristic responses as i) upregulation of pro-survival factor- glial cell lines-derived neurotrophic factor (GDNF), vascular endothelial growth factor (VEGF), BDNF, nerve growth factor (NGF), neurotrophin-3 (NT-3), p75^NTR^, N-cadherin, erythropoietin, ii) activation of innate immunity- upregulation of TNF-α, IL-1α, IL-1β, leukaemia inhibitory factor (LIF), MCP-1 in the distal part of the neuron, iii) LIF and IL-6 acts directly by attracting macrophages, iv) clearance of myelin debris and promoting vascularization of bridges formed between proximal and distal end (Fig. 2) (Cattin et al. 2015; Liu et al. 2019).

Injured neurons conduct a characteristic move to regain the axonal integrity by initially following the demyelination at the cut ends. This response proceeds with the WD resulting in apoptosis of the complete distal stump. As the degeneration proceeds, repair Schwann cells start forming the bridge and construct the axon from scratch. During the process, repair cells release IL-6 and (LIF) which accelerates the debris clearing and make the way for the regeneration of axonal fibres (Fig. 2).

Repair cells play a key role in structural repair following axonal growth. They act as the building blocks to reconstruct the axon. The reconstruction process proceeds from the distal end forming a bridge that joins the microtubular structure on the proximal end known as the growth cone. The process of WD is overlapping with the reconstruction phase. To clear the way for the newly developing axon, myelin and damaged fibres are eliminated with the help of repair cells or the repair Schwann cells (Jessen and Mirsky 2016; Jessen et al. 2015). These cells when activated, show upregulation of LIF and IL-6, these factors attract macrophages at the injured site eliminating myelin debris and damaged axons. Stimulated macrophages produce VEGF, which binds with the VEGF receptor on the endothelial cell surface. This interaction promotes neurogenesis and neovascularization of the bridge between the proximal and the distal stump (Cattin et al. 2015; Hu and Olsen 2017; Liu et al. 2019).

## Stimulatory Role of BDNF in Neuronal Distress

BDNF is one of the widely studied and explored proteins of the neurotrophin family. Neurotrophins are a class of proteins that are involved in the modulation of synaptic activity, neuronal survival and release of neurotransmitters. They are responsible for provoking plasticity and the growth within neurons of the peripheral and central nervous systems. Numerous studies conducted on traumatic injury models exhibit the role of neurotrophins in the repair and regeneration process in neurons. Hints to the diverse protective role of neurotrophins come from studies conducted in mutant animals. It was observed that knocking out a neurotrophin gene lead to mortal phenotypes. Neurotrophins like BDNF are even involved in the development of the nervous system. Transgenic mice lacking BDNF could hardly reach adulthood, disclosing developmental functions of neurotrophins (Ernfors et al. 1994; Jones et al. 1994). Clinically, plasma levels of BDNF are proposed as a potential biomarker for the assessment of the brain’s cognitive function and provide treatment efficacy (Levada et al. 2016). Various proteins like BDNF and GDNF mark their presence during the early stage of the regeneration process (Menorca et al. 2013). Neurotrophins guide the development of sprouting growth cones from the proximal stump by stabilizing the cytoskeleton for the reorganization of structural components. The mobility of these cones depends on receptor feedback or contact-dependent (chemo response) called Neurotropism. Some of the guidance molecules include somatotropins, epinins, netrins, and slits. Growth cones are inhibited by Collapsin-1 molecule, however, BDNF decreases the susceptibility to Collapsin-1 oriented degradation of growth cones aiding the regeneration process. Tissue scars are accounted as physical barriers for the regenerating growth cones. These scar tissues are countered and cleared with the help of proteases and plasminogen activators, providing unrestricted movement for growth cones. Concluding all the factors, the key points to neuronal regeneration are smaller gap distance, WD, axon guidance specificity, end-organ viability, intact endoneurium, lesser tissue scar and formation of bands of bungers (Jessen and Mirsky 2019; Kang and Lichtman 2013).

Although peripheral nerves have greater potential for regeneration, complete recovery and functionality is still a question. In such conditions, neurotrophins prove to be of great importance especially BDNF. It is a member of the neurotrophin family, which includes NT-3,4/5, NGF and BDNF (Huang and Reichardt 2001; Lou et al. 2005; McGregor and English 2019).

**Fig. 3:**
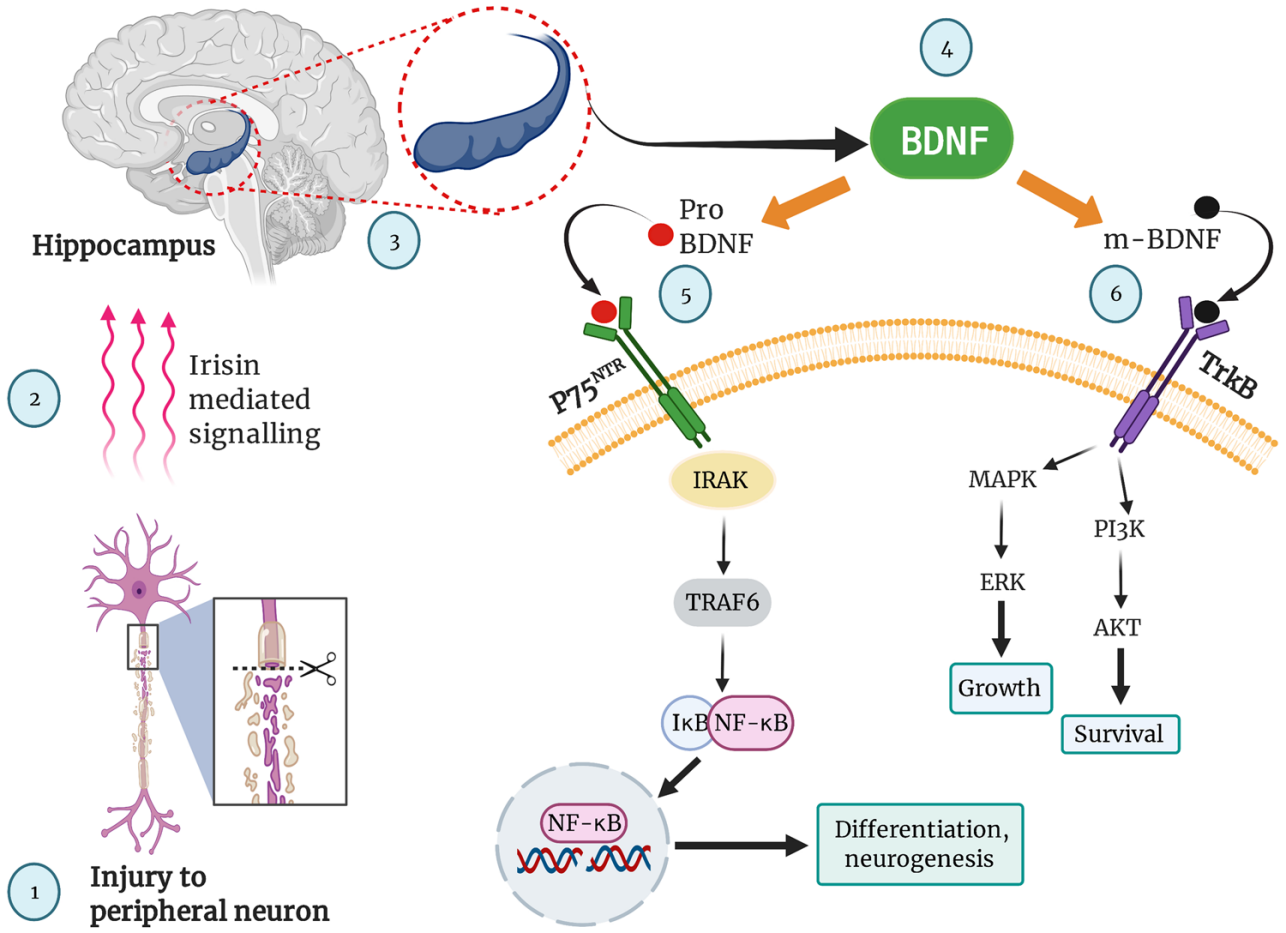
Schematic representation of BDNF mediated signalling and neuroprotective mechanism promoting cell survival. Injured peripheral neuron triggers cascade of events showcasing survival instinct and practising neuroplasticity by activating irisin mediated signalling (created with BioRender.com) Abbreviations: BDNF, p75 neurotrophin receptor (p75^NTR^), tropomyosin receptor kinase B (Trk-B), interleukin-1 receptor-associated kinase (IRAK), tumour necrosis factor receptor-associated factor 6 (TRAF6), an inhibitor of nuclear factor kappa B (IκB), nuclear factor kappa B (NF-κB), mitogen-activated protein kinase (MAPK), phosphoinositide 3-kinase (PI3K), extracellular signal-regulated kinase (ERK), protein kinase B (AKT). (created with BioRender.com)

Irisin mediated signalling stimulate the hippocampus to synthesize BDNF by increasing the expression of neurotrophin receptors (Fig. 3). BDNF is released as pro and mature (m-BDNF). Pro BDNF binds to p75^NTR^ on the cell surface leading to activation of IRAK and TRAF6 pathways. The m-BDNF is known to bind on the TrkB receptor found on the cell surface leading to activation of MAPK and PI3K pathway, consequently assisting neurogenesis and its survival. Among other neurotrophins, BDNF is the most studied with a large number of research papers establishing its functional role as a major neuroprotective for chronic CNS and PNS disorders. The gene for BDNF is found on chromosome number 11. It is synthesized in the endoplasmic reticulum (ER) of the hippocampus, and secretion is highly activity-dependent. BDNF is synthesized as a precursor protein (pro-BDNF), which proceeds via Golgi apparatus to transmembrane Golgi network (TGN), from here it is released from vesicles when triggered by activity. BDNF is proteolytically converted to mature BDNF from pro-BDNF by extracellular proteases like plasmin. Mature BDNF interacts with the TrkB receptor while the pro-BDNF interacts with p75^NTR^ on both pre and postsynaptic membranes (Huang and Reichardt 2001; Lessmann et al. 2003; Thomas and Davies 2005). Activation of p75^NTR^ results in the initiation of the pro-apoptotic cascade, however, this does not hold for all cell types. p75^NTR^ is generally downregulated or suppressed in adults but gets re-expressed under pathological conditions or cellular insults like axon damage or neurodegeneration. It plays an important role in the survival of neurons by acting opposite to their normal mechanism. Post nerve damage, neurons observe re-expression of quiescent p75^NTR^ receptor activated by pro-BDNF resulting in activation of NF-κB via IRAK mediated signalling. This cascade of events is responsible for the differentiation and neurogenesis of axon post damage (Goncharuk et al. 2020; Skeldal and Coulson 2016). BDNF plays a key role in the survival, growth and functionality of the neuron. It also plays a key role in conserving neuronal integrity and maintaining the synaptic plasticity of a nerve cell (An et al. 2008; Huang and Reichardt 2001; Thomas and Davies 2005). Experimental pieces of evidence prove BDNF can significantly affect the rate of axonal regeneration, myelination and aid functional recovery (Ohnishi et al. 1996). In a preclinical study (Ohnishi et al. 1996), 20 mg/kg of BDNF was administered subcutaneously to Sprague -Dawley rats for four weeks, where sciatic nerve showcased significant recovesry from experimental crush injury as compared to the injury control group. As per the study (Ohnishi et al. 1996), administration of BDNF significantly improved the recovery profile showing an increase in the total number, density and diameters of myelinated fibres. A mechanistic study (Zheng et al. 2016) was conducted on C57BL/6 J mice, in which the sciatic nerve was subjected to crush injury. The animals were treated with BDNF and anti-BDNF respectively, animals in the BDNF group showed better recovery and regeneration. Based on the results of microscopic evaluation and immunoblotting, BDNF promoted mRNA expression provoking intrinsic regeneration capacity of the neuron. It was also observed providing trophic support to the distal end of the neuron thereby preventing denervation.

## Diverse Role of PACAP

PACAP and BDNF are neurotrophins showing a vital role in neuroprotection and repair. PACAP was isolated from the ovine hypothalamus in 1989, which stimulates adenylate cyclase activity in the pituitary (Miyata et al. 1989). According to a study conducted on rats, PACAP showed neuroprotective action and aided cell survival in ethanol-induced toxicity of the cerebellum (Botia et al. 2011). Experimental sciatic nerve compression performed on rats characterised an increase in mRNA expression of PACAP in both dorsal root ganglion neurons and sciatic nerve (Pettersson et al. 2004). PACAP is found upregulated in response to inflammation, pain, axotomy and peripheral nerve compression. Upregulation of PACAP is detectable in motor neurons after about 6 h of peripheral damage, however, the maximal levels are obtained after about 48 h with an upregulation of about 20 times (Zhou et al. 1999). PACAP is a 27 or 38 amino acid peptide encoded by the ADCYAP1 gene located on human chromosome number 18, it is a member of the vasoactive intestinal polypeptide (VIP) family (Arimura et al. 1994; Hosoya et al. 1992). PACAP binds to PAC-1 receptor (G- protein-coupled receptor) and their signalling works by coupling to G- protein α subunits Gs and Gq consequently activating the characteristic adenylyl cyclase (AC) and phospholipase C (PLC) enzymes which in turn increases the synthesis of cAMP (cyclic adenosine monophosphate) and IP_3_ (inositol triphosphate).

**Fig. 4:**
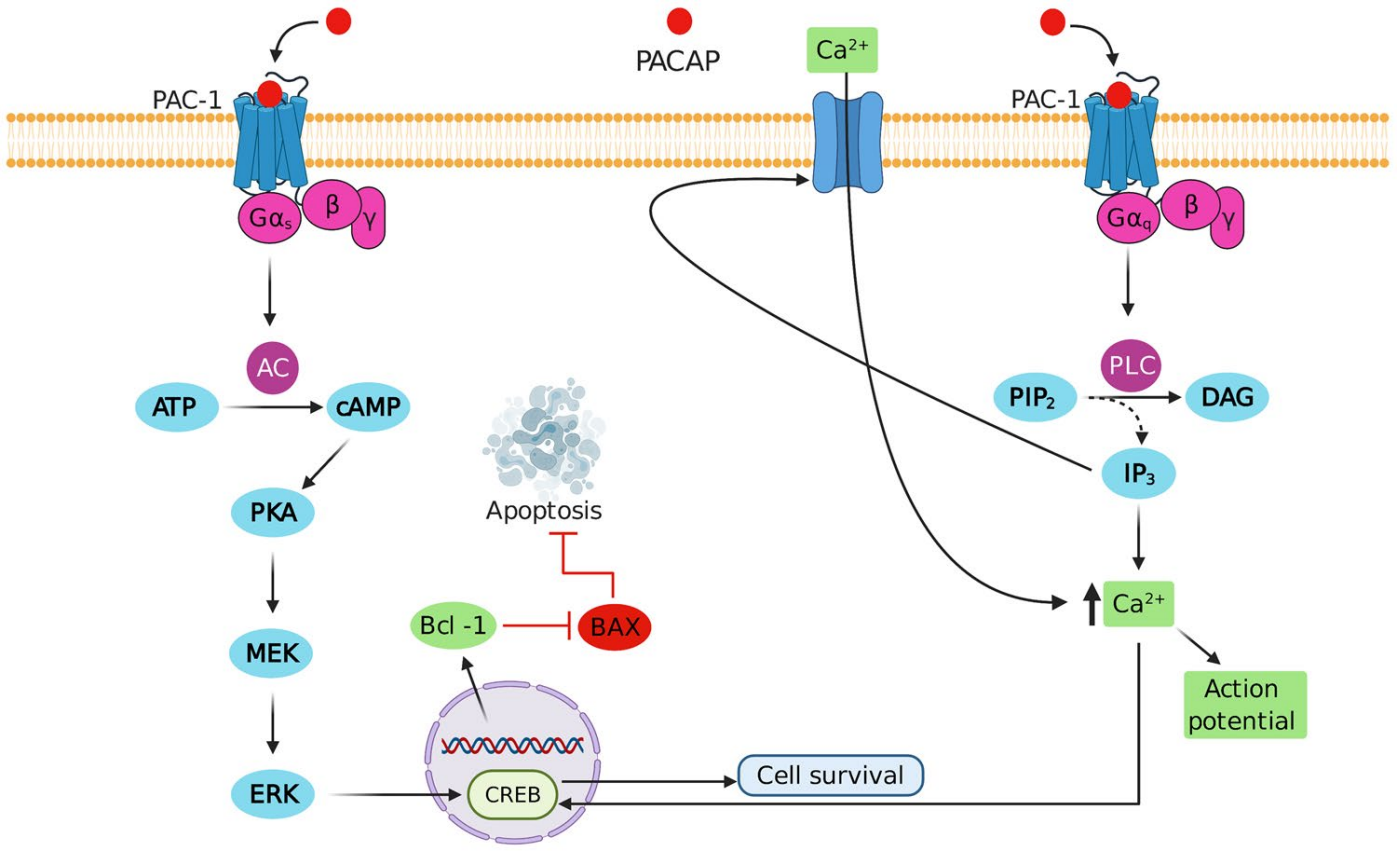
Schematic illustration representing dual neuroprotective action of PACAP. Abbreviations: pituitary adenylate cyclase-activating polypeptide type I receptor (PAC-1), adenylate cyclase (AC), adenosine triphosphate (ATP), cAMP (cyclic adenosine monophosphate), protein kinase A (PKA), mitogen-activated protein kinase (MEK), extracellular-signal-regulated kinase (ERK), cyclic adenosine monophosphate response element-binding protein (CREB), B-cell lymphoma 2 (Bcl-2), B-cell lymphoma 2 associated X protein (BAX), phospholipase C (PLC), phosphatidylinositol 4,5-biphosphonate (PIP_2_), diacylglycerol (DAG), inositol triphosphate (IP_3_). (created with BioRender.com)

The PACAP-PAC-1 complex acts through Gq and Gs subunits of the PAC-1 receptor. Interaction with these two subunits is marked with activation of secondary messengers i.e., AC and PLC leading to increases cAMP and IP_3_ synthesis. Activation of cAMP/PKA/MEK/ERK/CREB pathway results in increased levels of Bcl-1 proteins inhibiting BAX, which in turn terminates apoptosis. Activation of IP_3_ leads to increase Ca^2+^ influx aiding sustained action potential and activation of IP_3_/Ca^2+^/CREB pathway promoting cell survival. These secondary messengers as a result increase Ca^2+^ influx into the cell simulating action potential (AP). PACAP shows PKA (protein kinase A) dependent neuroprotective action by producing AP in burst patterns which are known to produce a neuroprotective effect (Bell and Hardingham 2011; Zorumski and Mennerick 2000). Introduction of PACAP to neurons trigger sustained AP bursts (Costa et al. 2009) and Ca^2+^ influx into the cell plays a vital role in protection against apoptotic based damage by activating CREB (cAMP response element-binding protein)-mediated gene expression (Fig. 4) (Baxter et al. 2011; Dickson and Finlayson 2009; Waschek et al. 2006). PACAP is found to be expressed in a wide range of physiological systems in humans. It is expressed in the cardiovascular system, renal/urinary system, gonadal system, gastrointestinal system, pancreatic system, eye, respiratory, thyroidal, adrenal, lymphoid and osteological system (Cardell et al. 1991; Gaytan et al. 1994; Józsa et al. 2018; Liao et al. 2019; Maugeri et al. 2018, 2019a, b; Mungan et al. 1992; Nakamura et al. 2014; Reglodi et al. 2018; Waschek et al. 2006; Xu et al. 2016). PACAP is known to promote cell growth and maturation of cortical neuroblast, cerebellar granule and cells of dorsal root ganglion during CNS development. It is also known to aid the maturation and functionality of microglial cells (Nakamachi et al. 2011). The presence of PACAP is noted in parasympathetic, sympathetic and sensory neurons of PNS. Several studies mark the presence of PACAP in Schwann cells as it is highly important in neural development and gets expressed whenever there is any insult or damage to the tissue (Maugeri et al. 2020; Vaudry et al. 2000; Waschek et al. 2006). A Study (Nakajima et al. 2013) conducted on cultured monkey trigeminal nerve cells confirmed the upregulation of PACAP-27 through PAC-1 receptor, causing PACAP-induced neurite growth. The outgrowth was a result of triggered PLC and AC, thereby increasing the intracellular levels of Ca^2+^. cAMP/MEK signalling acts as a key to the protective action of PACAP by activating ERK/CREB pathway. PACAP thereby exhibits antiapoptotic action in damaged neurons, protecting and preparing the nerve cells for regeneration (Castorina et al. 2008; Nakajima et al. 2013; Shioda and Nakamachi 2015; Zhou et al. 1999).

## Conclusion

End organ reinnervation/ complete functional recovery in peripheral nerve damage is still an ambitious phenomenon for the scientific community. This naturally justifies the need to refurbish the current therapy regime for nerve injury emphasizing more on combination therapy. Endogenous neurotrophins can prove to be the key to pharmacological intervention as activating the intrinsic regenerative potential of neurons may assist drug therapy to produce promising results in future by naturally accelerating the repair process. Being endogenous peptides, BDNF and PACAP are significantly bio-relevant revealing an extensive potential for future studies.
